# A Sensitivity Analysis Framework Using the Proxy Pattern–Mixture Model for Generalization of Experimental Results

**DOI:** 10.1002/sim.70313

**Published:** 2025-11-07

**Authors:** Rebecca R. Andridge, Ruoqi Song, Brady T. West

**Affiliations:** ^1^ Division of Biostatistics The Ohio State University College of Public Health Columbus Ohio USA; ^2^ Survey Research Center of the Institute for Social Research University of Michigan‐Ann Arbor Ann Arbor Michigan USA

**Keywords:** causal inference, generalizability, randomized trials, selection bias, transportability

## Abstract

Generalizing findings from randomized controlled trials (RCTs) to a target population is challenging when unmeasured factors influence both trial participation and outcomes. We propose a novel sensitivity analysis framework to assess the impact of such unmeasured factors on treatment effect estimates called the Proxy Pattern‐Mixture Model in the context of RCTs (RCT‐PPMM). By leveraging proxy variables derived from baseline covariates, our framework quantifies the potential bias in treatment effect estimates due to nonignorable selection mechanisms. The RCT‐PPMM relies on two bounded sensitivity parameters that capture the deviation from sample selection at random and that can be varied systematically to determine how robust trial results are to a departure from ignorable sample selection. The approach only requires summary‐level baseline covariate data for the target population (not individual‐level data), thus increasing its applicability. Through simulations, we demonstrate that RCT‐PPMM can provide information about the potential direction of bias and provide credible intervals that capture the true treatment effect under various nonignorable selection scenarios. We illustrate the use of the method using a yoga intervention RCT for breast cancer survivors, illustrating how conclusions may shift under plausible selection biases. Our approach offers a practical and interpretable tool for evaluating generalizability, particularly when individual‐level data on nonparticipants are unavailable, but summary‐level covariate data are accessible.

## 1 Introduction

Randomized controlled trials (RCTs) are the gold standard for evaluating treatments and interventions for cancer patients and survivors [1], and in the absence of missing data or noncompliance, they provide internally valid estimates of treatment effects. However, external validity—whether a trial's results can be generalized to a target population—is only guaranteed if the trial sample is a random probability sample from the specific target population [2]. Most clinical trials use convenience samples (e.g., of volunteers), not probability samples, and differences between the trial sample and the target population are likely to exist as a result. In fact, certain demographic groups are consistently underrepresented in cancer trials, including older patients, patients from racial minorities, patients from rural areas, and patients with lower socioeconomic status [3, 4, 5, 6, 7, 8, 9]. Trial participants have also been shown to tend to have fewer comorbidities but have more advanced disease [10].

To the extent that these factors—or other unmeasured characteristics—are associated with trial outcomes, a given trial may produce biased estimates of treatment effects in the broader population. Adjustment for observed differences between the sample and target population can provide unbiased treatment effects for the population, if these factors are measured in both the trial sample and the population [11]. However, these observed differences often do not fully explain the failure of trial results to replicate in a target population, often referred to as the efficacy–effectiveness gap [12, 13]. For example, several recent studies have shown poorer survival and smaller treatment effects in “real‐world populations” after treatments for small cell [14] and non‐small cell [15, 16] lung cancer than were shown in clinical trials. Importantly, differences in treatment effect sizes were not completely explained by differences in demographic composition between the trial samples and the real‐world populations. Other recent work has shown that failures of trial results to generalize are not due to inclusion/exclusion criteria alone [17].

These findings underscore the need to consider how *unmeasured* characteristics may differ between trial participants and the broader population, and the potential impact of these differences on how treatment effects from a trial generalize to a population outside the study sample. While there are certainly additional factors that may partially explain the efficacy‐effectiveness gap, such as differences in intervention delivery or treatments being offered to patients who do not match the original target population, we posit that considering unmeasured differences between who chooses to participate in a randomized trial and who does not may be an important contributor to this gap.

A crucial component of all generalizability methods is the availability of baseline covariate data for the target population. Such data allow for the comparison of a sample to a population, and adjustment for differences in composition. When assessing the generalizability of trials involving cancer patients and survivors, there are many high‐quality sources for target population data. Cancer registries such as the Surveillance, Epidemiology, and End Results (SEER) database or the American College of Surgeons National Cancer Database can be used to represent a broad population of survivors. Alternatively, large probability samples such as the National Health Interview Survey or the National Health and Nutrition Examination Survey contain samples from the subpopulation of survivors. More local data sources such as electronic health records data or publicly shared data from cohort studies can be useful in generalizing to more restricted populations, for example, generalizing to all patients in a particular health system (in contrast to those who participated in the trial). However, all of these sources are likely to contain only a limited set of baseline covariates (e.g., age, race/ethnicity, tumor characteristics) that may not fully capture the set of individual characteristics associated with both trial outcomes and how likely an individual is to enroll in a trial. Thus, methods that assess the possible impact of unmeasured effect modifiers are needed.

Assessment of the generalizability of a trial's results can be done before the trial begins (a priori) or after outcomes have been collected (a posteriori) [18]. While there are pros and cons to both approaches, a posteriori methods allow the potential for bias to be assessed separately for different trial outcomes (e.g., primary and secondary outcomes) and are the predominant generalization method currently in use. Most existing methods for generalizability assume that, conditional on baseline covariates, individuals in the trial sample and not in the trial sample (i.e., in a target population) are exchangeable, regardless of whether the method is outcome model‐based [11, 19, 20, 21], propensity score‐based [19, 22, 23, 24, 25, 26], or doubly robust [11, 19, 27]. In other words, these methods assume that the potential outcomes for an individual are independent of trial participation, conditional on baseline covariates, an assumption also referred to as an ignorable selection mechanism. This exchangeability assumption explicitly assumes that there are no unmeasured effect modifiers [25, 28], a condition alternatively referred to as independence of sample selection and treatment effect heterogeneity [28], or unconfounded sample selection [25, 29]. If unmeasured effect modifiers exist, then outcome models and propensity score models estimable from the observed data are inherently biased, as they omit these variables.

Importantly, the effect of unmeasured effect modifiers is *untestable* given the observed data. Thus, the most appropriate way of assessing their potential impact is through a sensitivity analysis, whereby untestable assumptions are made on the impact of omitted variables and the sensitivity of generalizability inferences to these assumptions is tested. Relatively few sensitivity analyses have previously been proposed to assess departures from the exchangeability assumption, and they have limitations. Existing methods are limited to continuous outcomes [30], or require observed data on the effect modifier in the sample (unobserved only for the population) [31, 32], or use multidimensional [33] or difficult to interpret [34] sensitivity parameters. Our goal was to develop methodology that overcomes these limitations and is easy to apply in practice.

In this paper we propose a novel, easily interpretable, sensitivity analysis framework for assessing the generalizability of RCT findings to a target population when unmeasured effect modifiers are present. In Section 2 we describe the methodology, and in Section 3 we illustrate the properties of the method via a simulation study. In Section 4.1 we demonstrate the use of the method by applying it to a two‐arm RCT that assessed whether Hatha yoga could improve inflammation and fatigue among breast cancer survivors relative to a wait‐list control [35]. Section 5 concludes with advice on using the method in practice, discussion of limitations, and areas for future work.

## 2 The Proxy Pattern‐Mixture Model for Assessing Generalizability of Treatment Effects in an RCT

The issue of RCT generalizability is ultimately one of selection bias. Specifically, are the individuals selected into the trial sample systematically different from individuals not in the trial sample (in the target population)? If so, then this is evidence of selection bias. Observable differences between the trial sample and target population (e.g., differences in demographic composition) can be “adjusted away” using existing generalizability methods and are the RCT equivalent of ignorable selection [36, 37], which is itself an extension of the idea of ignorable nonresponse first described in the missing data literature [38, 39]. If, however, there are factors that are associated with trial outcomes—effect modifiers—that are unobserved, then the result is nonignorable selection bias.

Our proposed approach is based on proxy pattern‐mixture models (PPMMs), which were first developed for assessing nonignorable nonresponse bias in surveys [40, 41, 42, 43, 44] and nonignorable missing data in large cancer databases [45]. These methods have since been extended to assess selection bias in nonprobability samples [46, 47, 48, 49]. These methods use a model‐based approach to develop estimates (means, proportions, regression coefficients) for key outcome measures when sample response or sample selection is nonignorable, that is, it depends to at least some extent on the outcome of interest (even after adjusting for observed covariates). We adapt this approach of using PPMMs to the problem of selection bias in the context of RCTs. PPMMs as previously implemented do not directly apply to the causal inference framework of RCTs, where under the counterfactual framework we have two outcomes in the case of binary treatment/control: the outcomes under each treatment type. We call our novel approach the RCT‐PPMM.

### 2.1 Data and Notation

An RCT has been conducted on a sample of n individuals, with the selection of participants assumed to be non‐random. We would like to use the results of the trial to estimate the treatment effect in the target population. Let S be the indicator for being in the trial sample (S=1 for participants randomly assigned to treatment or control, S=0 for nonparticipants, that is, individuals in the target population but not in the trial sample). If the trial participants are a subset of the target population and interest lies in estimating the treatment effect in the full population, this is called *generalizability*, whereas if the trial participants are external to the population and interest is in estimating the treatment effect in this external population this is called *transportability*. The method described can be applied in both cases.

We assume that there are two treatments to be compared, denoted a=0 for control and a=1 for intervention, and let A denote the treatment assignment for individuals in the trial (S=1). For each a, the random variable $Y^{a}$ denotes the potential (counterfactual) outcome of interest under treatment a. For a given trial participant we observe the study outcome, $Y=AY^{1}+\left(1-A\right)Y^{0}$, that is, the potential outcome for the assigned treatment, as well as baseline covariates Z. For nonparticipants (S=0) we do not observe either Y or A, but we assume that we have target population‐level information about Z available in aggregate (i.e., the mean vector and covariance matrix). This information could come from large databases (e.g., cancer registries) or as weighted estimates from large probability samples from the target population. We note that, for most trials, even when assuming the trial sample is a subset of the population the sampling fraction will be small (e.g., a moderate‐sized trial of breast cancer patients compared with the population of all breast cancer patients in the U.S.) and thus population‐level information from such sources can be used. Additionally, we assume that there may exist some *unmeasured effect modifiers* U which are associated with both sample selection (S) and the potential outcomes ($Y^{a}$) but that we do not observe these for either trial participants or nonparticipants. Our goal is to quantify the impact that the unobserved U might have on estimates of the population average treatment effect in the population of nonparticipants, $E\left[Y^{1}-Y^{0}|S=0\right]$. When the trial sample is a subset of the target population, the marginal treatment effect would also be of interest, $E\left[Y^{1}-Y^{0}\right]$, and this is easily estimated as a weighted average of $E\left[Y^{1}-Y^{0}|S=1\right]$ and $E\left[Y^{1}-Y^{0}|S=0\right]$.

We make the standard assumptions to enable identification of potential outcome means for the non‐trial population, which are common across existing methods for generalizability of trial results [11, 23, 25, 26, 50]. We assume the trial is internally valid (conditional ignorable treatment assignment; positivity of treatment assignment; mean exchangeability over A) and that there is positivity of trial participation, that is, Pr[S=1|Z=z]>0 for all units in the target population. Importantly, we do not assume conditionally ignorable sample selection, also called exchangeability over S; we aim to use a sensitivity analysis to measure the impact of deviation from this assumption on estimates of the treatment effect.

### 2.2 The RCT‐PPMM

Following our previously developed methods for nonresponse bias [40] and selection bias in non‐probability samples [46], we first regress $Y^{a}$ on Z using the trial data (S=1), separately for A=1 and A=0 (i.e., by treatment arm). Typically, these would be parametric models such as linear regression models, and as with all model‐based methods care should be taken to assess model fit. From these models, we calculate the predicted value of $Y^{a}$ from the regression model corresponding to the assigned treatment (a=0,1). We denote these predictions $X^{a}$ and call them the *proxies* for the potential outcomes, since they represent the “best” predictions of the outcomes within each treatment arm given the observed baseline covariates. For the nonparticipant population we do not have individual‐level data, but we can calculate the *average* proxy value ${\bar{X}}^{a}$ (and an estimate of its variance) under each treatment, since we have the average Z value for S=0 (from our population data source), by plugging the mean values of Z into the fitted regression function.

We now apply our previously developed *proxy pattern‐mixture model* [40] (PPMM) for continuous outcomes, separately by treatment arm, to estimate the potential outcomes means for nonparticipants under varying assumptions about the sample selection mechanism. Specifically, we assume that:

(1) $$\begin{array}{ll} & \left(Y^{a},X^{a}|A=a,S=j\right)∼N\left(\left(\begin{array}{c} \mu_{y\left(a\right)}^{\left(j\right)}\\ \mu_{x\left(a\right)}^{\left(j\right)} \end{array}\right),\right.\\ & \;\left.\left[\begin{array}{cc} \sigma_{\mathrm{yy}\left(a\right)}^{\left(j\right)} & \rho_{a}^{\left(j\right)}\sqrt{\sigma_{\mathrm{yy}\left(a\right)}^{\left(j\right)}\sigma_{\mathrm{xx}\left(a\right)}^{\left(j\right)}}\\ \rho_{a}^{\left(j\right)}\sqrt{\sigma_{\mathrm{yy}\left(a\right)}^{\left(j\right)}\sigma_{\mathrm{xx}\left(a\right)}^{\left(j\right)}} & \sigma_{\mathrm{xx}\left(a\right)}^{\left(j\right)} \end{array}\right]\right). \end{array}$$

Here, for treatment A=a, the joint distribution of outcome $Y^{a}$ and proxy $X^{a}$ is bivariate normal, with distinct means {$\mu_{y\left(a\right)}^{\left(j\right)},\mu_{x\left(a\right)}^{\left(j\right)}$}, variances {$\sigma_{\mathrm{yy}\left(a\right)}^{\left(j\right)},\sigma_{\mathrm{xx}\left(a\right)}^{\left(j\right)}$}, and correlation {$\rho_{a}^{\left(j\right)}$} for trial participants who received a specific treatment (*S* = 1) and nonparticipants (*S* = 1). In practice, this assumption of normality should be carefully checked by examining distributions of the outcome and predicted proxy values, possibly considering transformations of these values if necessary. In previous work we have shown that while mean estimates remain unbiased when the normality assumption is violated, variance estimates can be inflated [41]. Thus, appropriately transforming Y is an important step for inference.

The parameters of the PPMM for nonparticipants {$\mu_{y\left(a\right)}^{\left(0\right)},\sigma_{\mathrm{yy}\left(a\right)}^{\left(0\right)},\rho_{a}^{\left(0\right)}$} are not identifiable without additional assumptions, as these describe the distribution of the outcome $Y^{a}$ and its correlation with $X^{a}$ among nonparticipants. As has been previously described [40], these parameters are just identified by making an assumption about the sample selection mechanism, specifically that the probability a unit in arm A=a is in the trial sample is an unspecified function (f) of a linear combination of $X^{a}$ and $Y^{a}$:

(2) $$P\left(S=1|A=a\right)=f\left(\left(1-\phi_{a}\right)X^{a}/\sqrt{\sigma_{\mathrm{xx}\left(a\right)}^{\left(1\right)}}+\phi_{a}Y^{a},V\right),$$

where the division by the proxy's standard deviation is a mathematical convenience resulting in it having the same variance as the outcome. Here, V is a set of covariates orthogonal to $X^{a}$ and $Y^{a}$ but possibly associated with selection into the trial; this allows selection to depend on factors unassociated with the outcome.

The parameter $\phi_{a}$ is a sensitivity parameter that is not estimable from the data but instead should be varied to capture a range of assumptions about the selection mechanism. Values of $\phi_{a}$ range from 0 to 1 and describe the relative amount of selection that is due to $X^{a}$ and to $Y^{a}$. Specifically, if $\phi_{a}$ = 0, then selection is only a function of $X^{a}$ (and not $Y^{a}$), that is, an *ignorable* selection mechanism, which is also called *selection at random* and is the assumption made by the majority of existing generalizability methods. If, at the other extreme, $\phi_{a}$ = 1, then selection is only a function of $Y^{a}$ (and not $X^{a}$), that is, an “extreme” *nonignorable* selection mechanism, where trial participation depends entirely on the counterfactual outcome under treatment A=a. This is an unrealistic assumption but serves as a useful bound on the potential bias.

The targets of inference are the counterfactual means of $Y^{a}$ for nonparticipants (non‐selected units) for each treatment arm. Under the PPMM, the maximum likelihood estimate (MLE) of $\mu_{y\left(a\right)}^{\left(0\right)}$ for a given $\phi_{a}$ is a function of the outcome mean in the RCT (${\bar{y}}_{a}^{\left(1\right)}$), the proxy means for the trial sample (${\bar{x}}_{a}^{\left(1\right)}$) and non‐selected units (${\bar{x}}_{a}^{\left(0\right)}$), and the *strength of the proxy in the RCT*, that is, the estimated correlation between $X^{a}$ and $Y^{a}$ in the trial, ${\hat{\rho }}_{a}^{\left(1\right)}$:

(3) $${\hat{\mu }}_{y\left(a\right)}^{\left(0\right)}={\bar{y}}_{a}^{\left(1\right)}+\left(\frac{\phi_{a}+\left(1-\phi_{a}\right){\hat{\rho }}_{a}^{\left(1\right)}}{\phi_{a}{\hat{\rho }}_{a}^{\left(1\right)}+\left(1-\phi_{a}\right)}\right)\sqrt{\frac{{\hat{\sigma }}_{\mathrm{yy}\left(a\right)}^{\left(1\right)}}{{\hat{\sigma }}_{\mathrm{xx}\left(a\right)}^{\left(1\right)}}}\left({\bar{x}}_{a}^{\left(0\right)}-{\bar{x}}_{a}^{\left(1\right)}\right).$$

This follows from applying results previously derived for the PPMM to a single counterfactual outcome [40]. This estimate takes the difference in *proxy* means $\left({\bar{x}}_{a}^{\left(0\right)}-{\bar{x}}_{a}^{\left(1\right)}\right)$ for the non‐selected (target population) and selected (trial sample) units and uses this to shift the estimated outcome mean in the trial sample (${\bar{y}}_{a}^{\left(1\right)}$), after scaling by the standard deviations of the outcome and the proxy in the trial sample. This makes intuitive sense, since the proxy is the best predictor of $Y^{a}$ from the trial. The amount of shift is then scaled based on both ${\hat{\rho }}_{a}^{\left(1\right)}$ and $\phi_{a}$, where ${\hat{\rho }}_{a}^{\left(1\right)}$ captures how “good” of a proxy you have, that is, the strength of association between $Y^{a}$ and $X^{a}$ (and thus the strength of the baseline covariates Z in predicting $Y^{a}$). Note that if $\phi_{a}=0$, the resulting estimator reduces to ${\hat{\mu }}_{y\left(a\right)}^{\left(0\right)}={\bar{y}}_{a}^{\left(1\right)}+{\hat{\beta }}_{y.x\left(a\right)}^{\left(1\right)}\left({\bar{x}}_{a}^{\left(0\right)}-{\bar{x}}_{a}^{\left(1\right)}\right)$, where ${\hat{\beta }}_{y.x\left(a\right)}^{\left(1\right)}$ is the estimated slope using $X^{a}$ to predict $Y^{a}$ using the trial sample, which is the standard regression estimator under an ignorable selection assumption. This produces estimates for ${\hat{\mu }}_{y\left(a\right)}^{\left(0\right)}$ identical to those produced by *outcome model‐based* generalizability methods [19] that assume exchangeability over S. As such, we can vary $\phi_{a}$ away from 0 as a way of assessing the impact of loosening the exchangeability assumption.

The estimated mean treatment effect for the non‐selected (nonparticipant) units under the RCT‐PPMM framework is then given by

(4) $$\begin{array}{ll} {\hat{\mu }}_{y\left(1\right)}^{\left(0\right)}-{\hat{\mu }}_{y\left(0\right)}^{\left(0\right)} & ={\bar{y}}_{1}^{\left(0\right)}-{\bar{y}}_{0}^{\left(0\right)}+\left(\frac{\phi_{1}+\left(1-\phi_{1}\right){\hat{\rho }}_{1}^{\left(1\right)}}{\phi_{1}{\hat{\rho }}_{1}^{\left(1\right)}+\left(1-\phi_{1}\right)}\right)\\ & \;\sqrt{\frac{{\hat{\sigma }}_{\mathrm{yy}\left(1\right)}^{\left(1\right)}}{{\hat{\sigma }}_{\mathrm{xx}\left(1\right)}^{\left(1\right)}}}\left({\bar{x}}_{1}^{\left(0\right)}-{\bar{x}}_{1}^{\left(1\right)}\right)-\left(\frac{\phi_{0}+\left(1-\phi_{0}\right){\hat{\rho }}_{0}^{\left(1\right)}}{\phi_{0}{\hat{\rho }}_{0}^{\left(1\right)}+\left(1-\phi_{0}\right)}\right)\\ & \;\sqrt{\frac{{\hat{\sigma }}_{\mathrm{yy}\left(0\right)}^{\left(1\right)}}{{\hat{\sigma }}_{\mathrm{xx}\left(0\right)}^{\left(1\right)}}}\left({\bar{x}}_{0}^{\left(0\right)}-{\bar{x}}_{0}^{\left(1\right)}\right). \end{array}$$

This expression depends on a pair of sensitivity parameters, $\phi_{1}$ and $\phi_{0}$, and thus a sensitivity analysis based on the RCT‐PPMM approach requires selecting a pair of values for these two sensitivity parameters.

For ease of interpretation, we recommend allowing for the possibility that the selection is non‐ignorable for one of the arms while fixed as ignorable for the other. For example, if we hypothesize that a trial tends to recruit volunteers who may benefit more (in terms of their *potential* outcome) from an experimental drug, this corresponds to selection dependent on $Y^{1}$ (outcome under treatment). Thus setting $0<\phi_{1}<1$ and $\phi_{0}=0$ would be an appropriate approach. Alternatively, if we believe that individuals with “better” outcomes under placebo (e.g., larger $Y^{0}$) are more likely to self‐select into the trial, but that their potential outcomes under treatment are not a driver of participation, we would set $\phi_{1}=0$ and vary $\phi_{0}$ between 0 and 1. In this approach to the sensitivity analysis, selection either depends on the potential outcome under treatment or the potential outcome under control, but not both. Alternative combinations of $\left\{\phi_{1},\phi_{0}\right\}$ could be considered, for example, if we believe that individuals who have better outcomes under placebo *and* under control are self‐selecting into the trial, then we may want to set both sensitivity parameters greater than zero. However, in practice this often results in essentially a “canceling out” of the selection effect, in which the adjusted means under both treatment and control are shifted in the same direction, thus resulting in little change to the overall treatment effect estimates. This is illustrated in the data application in Section 4.1.

Importantly, this approach does not directly model how selection into the trial is related to unobserved effect modifier(s), but rather on how selection based on $Y^{a}$ would impact estimates of the treatment effect. This allows information in the trial data about the relationship between $Y^{a}$ and Z to inform the sensitivity analysis. For an unobserved effect modifier U to impact inference, it by definition must be associated with $Y^{a}$, and so an assumption that selection depends on $Y^{a}$ (i.e., $\phi_{a}>0$) is indirectly allowing for dependence on U.

Variance estimates for estimates of the treatment effect (4) obtained from the method above can be obtained via M‐estimation [51] for fixed $\phi_{a}$ values or Bayesian approaches that put a prior distribution on $\phi_{a}$. In our past work [46, 47, 48, 49], we have employed a fully Bayesian approach to making inference about parameters of interest based on the PPMM, beginning with random draws of the sensitivity parameters from a Uniform (0,1) distribution (given that no information is available in the observed data about these parameters). Assuming non‐informative Jeffreys priors for the parameters in the PPMM, this approach would then proceed with posterior draws of the means, variances, and covariances defined by the PPMM, along with corresponding posterior draws of the adjusted means in (3) [40]. Given draws of the adjusted means, one can then compute draws of the treatment effect as in (4), and compute credible intervals for this effect.

In general, we recommend using this Bayesian approach, especially when the trial sample size is small. The Bayesian computations for the RCT‐PPMM are straightforward when using conjugate priors and do not require any resampling methods, and thus are not computationally intense. In our application the Bayesian approach was much faster than the M‐estimation approach, owing to the need for a separate numerical optimization for every combination of fixed $\phi_{a}$ values for the M‐estimation approach. Putting a Uniform (0,1) prior on $\phi_{a}$ also reflects the lack of prior information in the data about this parameter and produces a single interval that reflects this uncertainty. In addition, in the Bayesian framework one could potentially incorporate (for example) imputation for sporadic missing data before applying the RCT‐PPMM.

## 3 Simulation Study

We conducted a simulation study to assess the ability of the RCT‐PPMM to provide information about the direction and magnitude of selection bias in a small, randomized trial using simulated data. Specifically, interest was in whether the RCT‐PPMM could provide information about the plausible direction of bias, and whether the suggested sensitivity analysis would cover the true treatment effect in the non‐selected sample (i.e., the set of units that did not participate in the trial). All simulations and data analyses were conducted in R [52] and the code is available at https://github.com/randridge/PPMA.

### 3.1 Data Generation

We generated populations of size N = 5000 containing a continuous outcome Y and three continuous covariates, {Z,W,U}, as follows. First, an observed baseline covariate was simulated as Z∼N(0,1) and an unobserved baseline covariate was simulated as U∼N(0,1), with Z⊥U. Then a third observed baseline covariate was simulated as $W∼N\left(\rho_{\mathrm{UW}}U,1-\rho_{\mathrm{UW}}^{2}\right)$ so that $\text{Corr}\left(W,U\right)=\rho_{\mathrm{UW}}$; the purpose of this covariate was to evaluate the impact of having partial information about the unobserved variable U via W. Potential outcomes were then generated as $Y^{0}∼N\left(Z+W+U,\sigma^{2}\right)$ and $Y^{1}∼N\left(1+\left(1+\gamma_{Z}\right)Z+\left(1+\gamma_{W}\right)W+\left(1+\gamma_{U}\right)U,\sigma^{2}\right)$ for all units in the population. Under this data generation model, the treatment effect in the full population ($E\left[Y^{1}-Y^{0}\right]$) is modified by Z if $\gamma_{Z}>0$, by W if $\gamma_{W}>0$, and by U if $\gamma_{U}>0$. The specific sets of these parameter values used were as follows: $\left\{\gamma_{U}=1,\;\gamma_{Z}=\gamma_{W}=0\right\}$ (effect modification by U only), $\left\{\gamma_{Z}=\gamma_{U}=1,\;\gamma_{W}=0\right\}$ (effect modification by Z and U), and $\left\{\gamma_{Z}=\gamma_{W}=\gamma_{U}=1\right\}$ (effect modification by all three covariates). The strength of the covariates in predicting the outcome was varied by varying $\sigma^{2}=\left\{1,4,13\right\}$; this parameter effectively determines the strength of the proxy in the RCT‐PPMM, that is, the values of $\rho_{a}^{\left(1\right)}$ in each arm. Smaller values of $\sigma^{2}$ correspond to stronger proxies, with the selected values of $\sigma^{2}$ corresponding approximately to $\rho_{a}^{\left(1\right)}$ values of {0.7, 0.55, and 0.4}, though these varied slightly by group and across different selection mechanisms. The correlation between W and U was varied as $\rho_{\mathrm{UW}}=\left\{0,0.3\right\}$. All combinations of these parameters were considered, resulting in 18 different data generation scenarios for the potential outcomes.

Once the potential outcomes were generated for a given simulated population, selection into a small, randomized trial was randomly simulated by calculating sampling probabilities using the following equation:

(5) $$\text{logit}\left(\mathrm{Pr}\left(S=1|Z,W,U\right)\right)=\beta_{0}+\beta_{Z}Z+\beta_{W}W+\beta_{U}U.$$

Five different parameter combinations were chosen to capture a range of possible nonignorable selection mechanisms, including an extreme nonignorable mechanism $\left(\beta_{U}=1,\beta_{Z}=\beta_{W}=0\right)$, partially nonignorable mechanisms with selection dependent on Z and U $\left(\beta_{U}=\beta_{Z}=1,\beta_{W}=0\right)$ or on all three covariates $\left(\beta_{U}=\beta_{Z}=\beta_{W}=1\right)$, and nonignorable mechanisms where selection was positively associated with the observed covariate(s) but negatively associated with the unobserved covariate: $\left(\beta_{U}=-1,\beta_{Z}=1,\beta_{W}=0\right)$, $\left(\beta_{U}=-1,\beta_{Z}=\beta_{W}=1\right)$. Only nonignorable mechanisms were considered, as existing methods that assume ignorable selection will produce unbiased estimates under this selection mechanism (including the RCT‐PPMM with $\phi_{1}=\phi_{0}=0$, which is identical to a regression model‐based generalizability approach). The intercept $\beta_{0}$ was chosen to produce a 5% sampling fraction, that is, so that the trial sample would be n=200 out of the population of size N=5,000. Samples of size n=200 were selected without replacement and with unequal sample selection probabilities calculated using Equation (4) using Brewer's sampling method [53]. Sampling in this manner (instead of, for example, using Bernoulli sampling) resulted in samples of exactly 200 for each replicate, thus reducing simulation error. The first 100 selected units (sorted by ID) were randomly assigned to the treatment arm (A = 1) and the remaining 100 units were assigned to the control arm (A = 0). The observed trial data then consisted of Z, W, and $Y=Y^{a}$ for selected units with A = a.

The required auxiliary data for the RCT‐PPMM consisted of the mean, variance, and covariance of Z and W for the non‐selected units (i.e., the 4800 units with S = 0). While microdata were simulated for the non‐selected units, they were discarded and only these summary statistics were retained in order to mimic the situation likely to occur in practice where only summary measures are available from large probability samples or administrative data sources. The unobserved effect modifier U was used for data generation only and then discarded; it was not used for estimation with the RCT‐PPMM. We note that this data generation and sample selection approach does not simulate data directly from the PPMM underlying our new methodology (Equations (1) and (2)), since the RCT‐PPMM assumes normality conditional on the selection indicator, while the data were generated from an unconditional normal distribution. Crossing the 18 potential outcome generation scenarios with the five selection mechanisms resulted in a total of 90 simulation scenarios. A total of 1000 replicates were run for each scenario.

### 3.2 Target of Analysis and Performance Measures

The estimand of interest was the treatment effect in the non‐selected sample, that is, $E\left[Y^{1}-Y^{0}|S=0\right]$. For each simulated data set we calculated the true treatment effect in the non‐selected sample as the average of the difference in the potential outcomes for the non‐selected units, and the trial estimate as the difference in the mean observed outcome in the treatment group (A = 1) minus the mean observed outcome in the control group (A = 0) using the selected sample. We also computed the trial estimate as the difference in group means for each simulated data set. We then computed MLEs for the RCT‐PPMM estimate of the treatment effect in the non‐selected sample for two combinations of the sensitivity parameters: selection dependent on the potential outcome under treatment, $\left\{\phi_{1}=\left\{0,0.5,1\right\},\phi_{0}=0\right\}$, and selection dependent on the potential outcome under control, $\left\{\phi_{1}=0,\phi_{0}=\left\{0,0.5,1\right\}\right\}$. We also calculated Bayesian estimates under the RCT‐PPMM that paralleled these MLEs by (1) setting $\phi_{0}=0$ and putting a Uniform (0,1) prior on $\phi_{1}$ for selection dependent on the potential outcome under treatment, and (2) setting $\phi_{1}=0$ and putting a Uniform (0,1) prior on $\phi_{0}$ for selection dependent on the potential outcome under control. In addition, we also replaced the Uniform (0,1) priors with Uniform (0,0.5) priors to capture an assumption of a “moderately” nonignorable selection mechanism. When selection depends only on U, we would expect this method to not cover the true treatment effect, but it may perform adequately when selection depends on both U and Z (or W) and would have shorter credible intervals and thus potentially be more useful in practice.

In assessing performance, our main goal was to determine whether the sensitivity analysis using the RCT‐PPMM would correctly identify the direction of bias in the trial estimate of the treatment effect, defined as

$$\text{Bias of Trial Estimate}=E\left[Y^{1}-Y^{0}|S=1\right]-E\left[Y^{1}-Y^{0}|S=0\right],$$

and whether the true treatment effect for the non‐selected sample, $E\left[Y^{1}-Y^{0}|S=0\right]$, would be contained in intervals based on the RCT‐PPMM. Thus, the primary performance measure was the nominal coverage of MLE intervals constructed with endpoints $\left\{\phi_{1}=0,\phi_{0}=0\right\}$ and either $\left\{\phi_{1}=1,\phi_{0}=0\right\}$ (assumes selection depends on the outcome under treatment) or $\left\{\phi_{1}=0,\phi_{0}=1\right\}$ (assumes selection depends on the outcome under control) and of 95% credible intervals from the Bayesian approach. We note that the MLE intervals are *not* confidence intervals; the endpoints of these intervals are the MLE point estimates as described above, meaning that they provide users with a range of plausible values given extreme choices of the sensitivity parameters. We also calculated the mean estimates (across replicates) for each combination of $\left\{\phi_{1},\phi_{0}\right\}$ using the MLE approach to visualize the resulting sensitivity analysis. For comparison purposes in the visualization, we also constructed a 95% confidence interval for the trial estimate (for each data generation/selection scenario) using the average standard error across replicates.

### 3.3 Results

**FIGURE 1:**
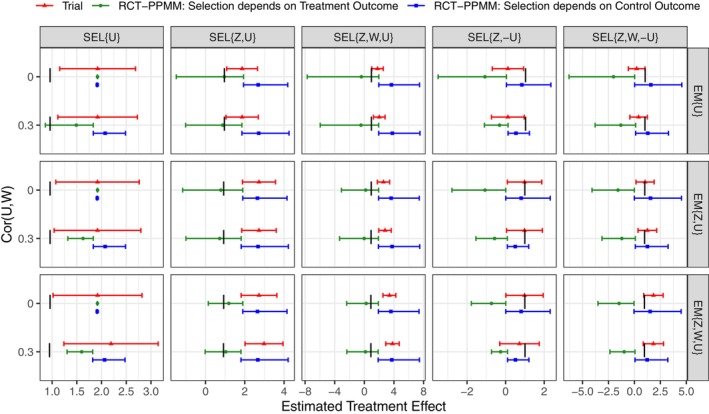
Empirical mean maximum likelihood estimates (MLEs) of the treatment effect under the RCT‐PPMM when there is a moderately strong proxy ($\sigma^{2}=4$). Columns correspond to different selection (SEL) mechanisms and rows correspond to different effect modification (EM) scenarios, where selection/effect modification always depends on an unobserved effect modifier U and may also depend on observed covariates Z and/or W. True treatment effect is shown with a black vertical line, trial estimate and 95% confidence interval (constructed using the average standard error across replicates) is shown with a red triangle, and RCT‐PPMM intervals based on the MLE point estimates are shown in green ($\phi_{1}=\left\{0,0.5,1\right\},\phi_{0}=0$) and blue ($\phi_{1}=0,\phi_{0}=\left\{0,0.5,1\right\}$).

As an illustration of how the RCT‐PPMM provides a sensitivity analysis, Figure 1 shows the empirical mean MLEs of the treatment effect under the RCT‐PPMM for the 30 data generation scenarios where there is a moderately strong proxy $\left(\sigma^{2}=4\right)$. In nearly all data generation scenarios, the trial estimate (red triangle) is badly biased, with positive bias when trial selection depends positively on the unobserved effect modifier U, and negative bias when trial selection depends negatively on U. However, the sensitivity analysis with the RCT‐PPMM captures the true treatment effect (black line) in most scenarios. Specifically, when selection is positively associated with U (first three columns), the RCT‐PPMM interval corresponding to selection depending on the outcome under treatment (green interval: $\left\{\phi_{1}=\left\{0,0.5,1\right\},\phi_{0}=0\right\}$) produces smaller estimates than the trial, that is, the RCT‐PPMM estimates are closer to the truth. Conversely, when selection is negatively associated with U (last two columns), the RCT‐PPMM interval corresponding to selection dependent on the outcome under control (blue interval: $\left\{\phi_{1}=0,\phi_{0}=\left\{0,0.5,1\right\}\right\}$) captures the true treatment effect.

A key feature of the RCT‐PPMM is that the pair of RCT‐PPMM intervals start from the same place and move in opposite directions. That starting point corresponds to ignorable selection, that is, $\phi_{1}=\phi_{0}=0$. This is the same estimate as obtained via outcome model‐based generalizability methods (specifically, using regression adjustment to obtain a covariate‐adjusted treatment effect). There is no information in the data to inform which interval is the “right” one. Thus, a reasonable sensitivity analysis would look at both intervals and determine what combination of parameters ($\phi_{1},\phi_{0}$) produce a null (zero) treatment effect, also known as a “tipping point” analysis [54]. Then one can assess in what direction and how severe the nonignorable selection would have to be (i.e., what value of $\phi_{a}$) to produce that null (zero) treatment effect, that is, to substantially change the conclusion drawn from the trial. This type of sensitivity analysis has previously been referred to as a “killer confounder” or “killer moderator” analysis [54]. We later illustrate this approach in our application.

Not surprisingly, when selection into the trial depends only on U (first column of Figure 1) and there is no information about U via a correlated covariate W available, the RCT‐PPMM cannot recover any information. The resulting zero‐width intervals would be an indication that there is no information in the observed data with which to conduct the RCT‐PPMM analysis. Fortunately, when there is at least some information available about U via a correlated covariate W, the RCT‐PPMM does in fact move estimates in the corresponding correct direction (i.e., towards the truth), and we see that only in the unusual case of both selection and effect modification depending only on U would the RCT‐PPMM completely fail.

When the proxy is stronger $\left(\sigma^{2}=1\right)$, meaning observed covariates are more strongly associated with trial outcomes among the trial participants, the pattern or results remains the same, but with shorter intervals. Similarly, when the proxy is weaker $\left(\sigma^{2}=13\right)$, the intervals are wider. Results for a weak proxy and a strong proxy are shown in Figures S1 and S2.

Coverage of the RCT‐PPMM MLE intervals and Bayesian intervals for a moderately strong proxy is summarized in Table 1 (coverage for weak and strong proxies is in Tables S1 and S2). As expected, when selection depends only on U, coverage of all three types of intervals is well below nominal; as was seen with the MLE estimates, the RCT‐PPMM does not have information with which to correct selection bias and thus coverage is low. In scenarios when selection depends on U and at least one observed covariate, the corresponding RCT‐PPMM Bayesian intervals with a Uniform (0,1) prior (allowing for a full range of $\phi_{a}$ values for one of the treatment arms) achieve nominal coverage (or greater). As with the MLE point estimates shown in Figure 1, the RCT‐PPMM analysis that assumes selection depends on the potential outcome under treatment results in at least nominal coverage when selection depends positively on U, and the RCT‐PPMM analysis that assumes selection depends on the potential outcome under control results in at least nominal coverage when selection depends negatively on U.

**TABLE 1:** Empirical coverage of intervals based on the RCT‐PPMM for the simulation study when there is a moderately strong proxy ($\sigma^{2}=4$).

			RCT‐PPMM: assume selection depends on treatment outcome			RCT‐PPMM: assume selection depends on control outcome		
Selection depends on	Effect modification by	Corr (U, W)	MLE	Bayes/Unif (0,1)	Bayes/Unif (0,0.5)	MLE	Bayes/Unif (0,1)	Bayes/Unif (0,0.5)
U	U	0	7.2	47.9	27.8	3.1	36.9	24.7
		0.3	54.8	91.0	58.1	0.7	21.1	25.8
	Z, U	0	2.0	38.1	26.4	3.1	36.7	25.5
		0.3	23.3	74.2	48.0	0.7	22.6	26.7
	Z, W, U	0	1.2	33.9	25.7	3.1	36.6	25.2
		0.3	21.0	73.0	49.6	0.7	22.2	26.4
Z, U	U	0	92.3	**99.4**	91.1	2.7	24.4	34.2
		0.3	**93.8**	**99.8**	93.1	3.5	27.5	37.4
	Z, U	0	**96.9**	**99.9**	92.7	2.7	25.0	34.3
		0.3	**96.2**	**99.8**	**95.3**	3.5	27.2	37.2
	Z, W, U	0	83.3	**98.6**	82.8	2.7	25.2	35.0
		0.3	88.3	**99.6**	87.0	3.5	27.8	38.5
Z, W, U	U	0	**95.1**	**98.9**	**99.3**	4.9	20.5	32.4
		0.3	93.3	**98.8**	**99.7**	6.7	23.8	35.6
	Z, U	0	**95.1**	**99.1**	**99.0**	4.9	21.6	34.4
		0.3	93.3	**99.2**	**99.0**	6.7	25.0	37.1
	Z, W, U	0	**95.1**	**98.8**	**98.2**	4.9	22.5	35.2
		0.3	93.2	**99.1**	**98.6**	6.7	25.7	37.9
Z, U	U	0	1.5	20.6	29.7	93.3	**99.9**	86.5
		0.3	2.4	46.5	47.8	56.6	**94.9**	76.7
	Z, U	0	1.5	15.1	25.5	93.3	**99.9**	86.3
		0.3	2.4	29.8	38.9	56.6	**95.1**	77.2
	Z, W, U	0	1.5	20.1	30.4	93.3	**99.8**	85.5
		0.3	2.3	38.2	44.6	56.6	**94.7**	76.4
Z, W, U	U	0	4.2	19.8	31.4	**95.7**	**99.1**	**97.2**
		0.3	4.6	22.5	34.2	**94.8**	**99.3**	**95.5**
	Z, U	0	4.2	21.6	32.9	**95.7**	**99.4**	**97.6**
		0.3	4.6	21.8	33.4	**94.8**	**99.6**	**94.6**
	Z, W, U	0	4.2	21.5	32.2	**95.7**	**99.3**	**97.3**
		0.3	4.6	23.9	36.0	**94.8**	**99.3**	**95.0**

*Note:* Bold denotes empirical coverage at or above 95% (accounting for Monte Carlo simulation error).

The MLE intervals (constructed as the interval between point estimates at the extreme values of the sensitivity parameters) have lower coverage rates than the Bayesian intervals. This is expected, as they fail to account for uncertainty in the construction of the proxies. The Bayesian intervals with the truncated Uniform (0,0.5) prior also have lower coverage than those with the Uniform (0,1) prior. However, they do show close to nominal coverage in many scenarios, despite effectively placing a cap on the extent of the non‐ignorable selection. Given these results, we would encourage the use of the Uniform (0,1) prior, consistent with our previous work in this area [46, 47, 49].

## 4 Application to a Yoga Intervention RCT

We applied our method to a completed RCT of breast cancer survivors that assessed whether Hatha yoga could improve inflammation, fatigue, and distress among breast cancer survivors (stage 0–IIIa) relative to a wait‐list control [35]. A total of 200 women were randomized to either participation in 12 weeks of yoga intervention or a waitlist control. Outcomes were measured at the end of the 12‐week period and again 3 months post‐treatment. Key behavioral outcomes included fatigue as measured by the total fatigue score on the Multidimensional Fatigue Symptom Inventory‐Short Form (MFSI‐SF) [55] and vitality as measured by the Medical Outcomes Study 36‐item short‐form health survey (SF‐36) [56]. At 3 months post‐treatment, women in the yoga group had significantly lower fatigue and higher vitality [35].

We applied our newly developed sensitivity analysis to assess the generalizability of these results to the pool of all eligible women at the study site at the time of the trial who did not participate in the trial (*n* = 1437). For analysis, we used the subset of trial participants with the key outcomes available at 3 months (*n* = 179; 87 control, 92 intervention). Covariate information for nonparticipants was limited to demographic and clinical information available in the medical record; specifically, we used age, race (white, Black, all other races), cancer stage (0, I, IIA, IIB, IIIA), and treatment type (chemotherapy, radiation, both, neither) to create the proxies.

For each outcome variable, we applied the RCT‐PPMM using the Bayesian approach to estimate the potential impact of nonignorable sample selection on the positive trial results. Two separate analyses were performed for each outcome to reflect the two possible selection mechanisms: (1) selection dependent on the control outcome $\left\{\phi_{1}=0,\phi_{0}∼\text{Uniform}\left(0,1\right)\right\}$, and (2) selection dependent on the treatment outcome $\left\{\phi_{1}∼\text{Uniform}\left(0,1\right),\phi_{0}=0\right\}$. Each analysis used 10 000 posterior draws.

**FIGURE 2:**
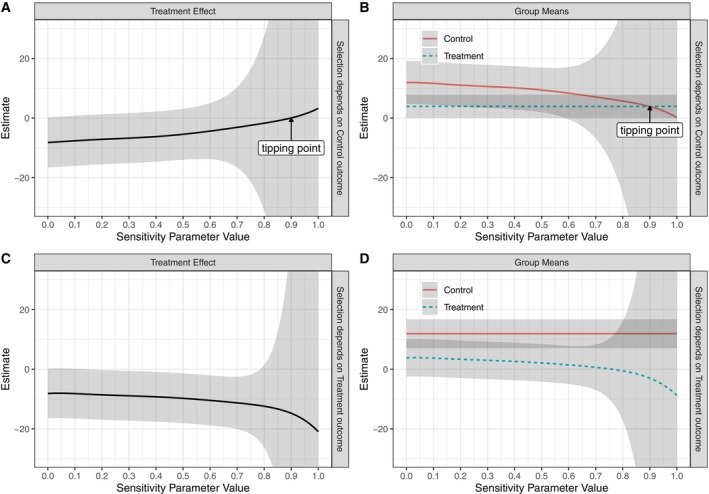
Bayesian estimates under the RCT‐PPMM for the fatigue outcome (MFSI‐SF). Panels A and B show the estimated treatment effect (A) and group means (B) when selection depends on the control outcome $\left\{\phi_{1}=0,\phi_{0}∼\text{Uniform}\left(0,1\right)\right\}$; Panels C and D show the estimated treatment effect (C) and group means (D) when selection depends on the treatment outcome $\left\{\phi_{1}∼\text{Uniform}\left(0,1\right),\phi_{0}=0\right\}$. Results are based on 10 000 posterior draws and shaded areas show 95% credible intervals.

**FIGURE 3:**
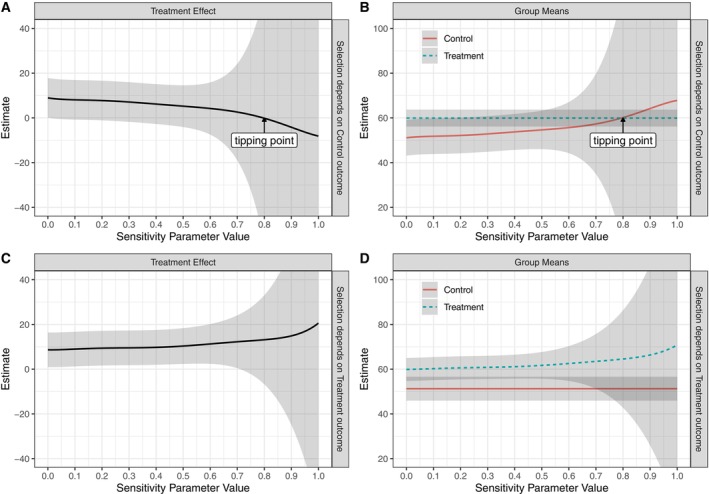
Bayesian estimates under the RCT‐PPMM for the vitality outcome (SF‐36). Panels A and B show the estimated treatment effect (A) and group means (B) when selection depends on the control outcome $\left\{\phi_{1}=0,\phi_{0}∼\text{Uniform}\left(0,1\right)\right\}$; Panels C and D show the estimated treatment effect (C) and group means (D) when selection depends on the treatment outcome $\left\{\phi_{1}∼\text{Uniform}\left(0,1\right),\phi_{0}=0\right\}$. Results are based on 10 000 posterior draws and shaded areas show 95% credible intervals.

Figures 2 and 3 present the estimated treatment effect and group means under the RCT‐PPMM, along with 95% credible intervals, for fatigue and vitality, respectively. For both outcomes, the top panels show selection dependent on the control outcome and the bottom panels show selection dependent on the treatment outcome. As was seen in the simulation study, the estimated treatment effect moves in opposite directions (i.e., towards zero versus away from zero) under these two different assumptions on the selection mechanism. Of particular interest are two types of “tipping points”: the value of the sensitivity parameter where the estimated treatment effect crosses zero, and the value of the sensitivity parameter where the credible interval for the treatment effect includes zero [54].

For fatigue, the estimated treatment effect under ignorable selection {$\phi_{1}=0,\phi_{0}=0$} is negative, consistent with the trial results where women in the yoga group had lower fatigue. As seen in panel (A) of Figure 2, when selection depends increasingly on the control outcome (as the sensitivity parameter value increases above 0), the treatment effect decreases, that is, approaches zero. In panel (B) we see that this is due to the estimated control mean becoming smaller as the nonignorable selection increases in strength. Importantly, the control group mean decreases but does not become implausible based on the limits of the fatigue scale (MFSI‐SF scores can range from −24 to 96).

The estimated treatment effect crosses zero at about $\phi_{0}=0.9$, which is a very strong nonignorable mechanism; therefore, in terms of direction of effect, this outcome appears robust to a nonignorable selection mechanism. In other words, the treatment effect goes to zero only when whether an individual participates in the trial is strongly dependent on their outcome under control. However, the credible intervals cross zero very close to ignorability; these intervals are relatively large due to the small sample size (*n* < 200), especially for large values of the sensitivity parameter. The bottom panels of Figure 2 show estimates when selection depends on the treatment outcome. In this case, the treatment effect increases in magnitude as selection depends more strongly on the treatment outcome (as $\phi_{1}$ increases above 0), driven by a decreasing mean for the treatment group (panel D).

The results for vitality follow a similar pattern, with a positive treatment effect under ignorable selection that moves towards zero as selection becomes dependent on control (Figure 3A). The estimated group means under the RCT‐PPMM remain plausible, given the range of the SF‐36 scale (0 to 100). The tipping point where the treatment effect goes to zero is at approximately $\phi_{0}=0.8$ (a strong nonignorable mechanism), indicating a robust effect. As with fatigue, the credible intervals cross zero very close to ignorability when selection depends on the control outcome, reflecting imprecise estimates due to the small trial sample size.

An important feature of this analysis is how the width of the credible intervals varies as a function of the sensitivity parameters. This is partially due to the weak proxies for the RCT‐PPMM. For fatigue, the proxy strengths in each arm are ${\hat{\rho }}_{1}^{\left(1\right)}=0.39$ and ${\hat{\rho }}_{0}^{\left(1\right)}=0.38$, and for vitality they are ${\hat{\rho }}_{1}^{\left(1\right)}=0.36$ and ${\hat{\rho }}_{0}^{\left(1\right)}=0.32$. With weak proxy strengths, the RCT‐PPMM results in drastically wide intervals for large values of $\phi_{a}$, consistent with the behavior of the PPMM as originally developed [40, 46].

In addition to the Bayesian approach, we obtained MLEs under the RCT‐PPMM for various combinations of the sensitivity parameters. The results were similar to the Bayesian analysis in terms of where the tipping points were and how intervals grew in size as the sensitivity parameter values increased towards one. Results are shown in Figures S3 and S4.

As described earlier, it could be possible for both $\phi_{1}$ and $\phi_{0}$ to be larger than zero, that is, for the selection mechanism to depend on both potential outcomes. One scenario would be to consider equal dependence on the two potential outcomes, that is, assume $\phi_{1}=\phi_{0}=\phi >0$. In this case, under the RCT‐PPMM with a Uniform (0,1) prior on the sensitivity parameter (taken to be the same for both arms), the resulting adjusted control group mean (as a function of ϕ) would be the same as that when selection depends on control, and the adjusted treatment group mean (as a function of ϕ) would be the same as when selection depends on treatment. In other words, the resulting estimates would be the control group line in Figure 2B and the treatment group line in Figure 2D (and similar for Figure 3). Since these means move in the same direction as ϕ increases (group means decrease for fatigue and increase for vitality), the resulting treatment effect will be relatively constant as a function of ϕ. This illustrates the “canceling out” of the selection effect under the RCT‐PPMM. The group means and resulting treatment effects under this equal ϕ scenario are shown in Figure S5.

## 5 Discussion

In this paper, we have described and evaluated a principled, model‐based, sensitivity analysis approach for assessing the generalizability of estimated treatment effects to a target population when unmeasured effect modifiers are present. An empirical simulation study demonstrates the ability of the proposed RCT‐PPMM approach to produce adjusted estimates of treatment effects that move in the direction of the known true treatment effect, given that an auxiliary proxy of the outcome of interest provides at least some information about the unmeasured effect modifier. Consistent with our prior work on measures of non‐ignorable selection bias, we find evidence of good performance of a fully Bayesian approach that places a Uniform (0,1) prior on the sensitivity parameter $\phi_{a}$ that governs the extent of the non‐ignorable selection in one of the two treatment arms, being allowed to very between 0 and 1 for one arm (while the other arm has this parameter fixed to 0). This approach allows analysts to consider a full range of sensitivity analyses under different selection mechanisms and ascertain what extent of non‐ignorable selection (and in which arm) would yield substantially different estimates of the treatment effect relative to the selected sample.

A subsequent application of the proposed methodology to a real data set from an actual RCT evaluating a Hatha yoga intervention for breast cancer survivors revealed the extent of non‐ignorable selection that would ultimately produce null treatment effects. In the case of this RCT, the selected sample of volunteers suggested that the yoga treatment had a significant negative effect on fatigue and a significant positive effect on vitality. Interestingly, the application suggested that non‐ignorable selection was most critical for the control arm of the RCT. That is, if the RCT tended to recruit volunteers who would tend to experience more fatigue and less vitality if *not* given the yoga treatment (in terms of their *potential* outcomes), the observed effect may have been overstated relative to what would be seen in a more general population.

This application engendered suggestions for how this type of sensitivity analysis should be interpreted in practice. A natural question is what sensitivity analysis scenario (some form of non‐ignorable selection based on the outcome under treatment or under control) is the best fit for evaluating the results from a given RCT. If the sensitivity analysis suggests that a plausible selection mechanism could in fact alter conclusions from the RCT significantly, we would recommend the following assessments for trying to understand whether that mechanism is likely:

1. If contact information is available for a small population and following up with individuals who did not express interest in the trial is feasible, attempt to collect data from a small sample of non‐participants on a very small set of key outcomes measured in the trial. Compare these outcomes to baseline outcomes in the control arm and see if significant differences emerge.
2. Leveraging the “continuum of resistance” theory from the survey nonresponse literature [57, 58, 59, 60, 61, 62, 63], where respondents with higher predicted response propensity are viewed as “likely respondents” and respondents with lower predicted response propensity are viewed as “likely nonrespondents”, use dates of expressing interest in the trial (e.g., signing up on a web page) to identify eager participants who expressed early interest (“participants”), and compare these participants to those participants who were potentially more cautious and signed up later (“non‐participants”) in terms of their outcomes (by treatment arm). Examine whether these analyses suggest potential selection as a function of the outcome of interest in either arm.
3. As illustrated in the application, in addition to evaluating the adjusted treatment effects, also conduct analyses of the adjusted estimates of the means in each arm (based on the PPMM). Examine whether the adjusted estimates of the means corresponding to an adjusted estimate of the treatment effect (for the values of the sensitivity parameters that are identified as resulting in a significant change in the treatment effect) are *scientifically plausible*. In simple cases, this may just involve determining whether the predicted mean (in either arm) associated with that type of adjustment is crossing plausible bounds for a scale (as in the application). In more complex cases, subject matter expertise may be needed to evaluate these types of tipping points [30], where the predicted means in each arm that are associated with the adjusted treatment effect in question (resulting, possibly, in a null effect) are not scientifically plausible.

We feel that attempting to follow these concrete steps and gauging the plausibility of sensitivity analysis scenarios that result in significant changes to conclusions from the observed RCT will be useful supplements to our proposed approach.

Our RCT‐PPMM approach offers important advantages over other proposed approaches for examining the effects of non‐ignorable selection on estimated treatment effects. Relative to the “bias functions” proposed by Dahabreh et al. [30], our approach is more data‐driven, and we can also write the RCT‐PPMM estimator in the context of their proposed framework (see Supporting Information). Assuming that the underlying PPMM is well specified, our proposed approach also eliminates the inefficiency in design‐based estimates of population treatment effects that arises from applying population weights to selected samples before estimating treatment effects (e.g., [26, 64]), where the use of weights in estimation increases the standard errors of the estimated treatment effects.

As illustrated in the simulation study, there are instances when the RCT‐PPMM will not be a useful approach. Since the RCT‐PPMM is data‐driven, its adjustments are based on observed differences between the proxy mean for trial participants (selected units) and the proxy mean for the target population (non‐selected units). In some cases, there may be no observed difference, or this difference will be very small. This will occur if selection does not depend at all on observed variables, and the resulting RCT‐PPMM estimates will not vary as a function of $\phi_{a}$, as was seen in the simulation when selection depended only on the unobserved variable U.

When this occurs, researchers would need to explore alternative sensitivity analyses. For example, if the RCT‐PPMM produces estimates that do not vary as a function of $\phi_{a}$, alternative sensitivity analysis approaches that assess the impact of violations of exchangeability include the bias function approach of Dahabreh et al. [30], the bounding approach of Chan [33], or the weighting approach of Huang [54]. All of these methods do not rely on observed differences between trial participants and non‐participants in the way the RCT‐PPMM does and thus would be reasonable alternatives when the RCT‐PPMM fails to produce informative results. In both the Dahabreh et al. and Chan approaches, the sensitivity parameter is the hypothesized difference in potential outcomes for trial participants and non‐participants, which could be chosen to be arbitrarily large (as opposed to under the RCT‐PPMM, where the magnitude of this difference is driven by the difference in proxy means and the correlation between the proxy and the outcome in the trial sample). In the Huang approach, sensitivity parameters are defined as correlations between an unobserved effect modifier and (separately) the treatment effect and the selection mechanism, and thus these could be specified even in the absence of observed covariates that are associated with selection.

One potential drawback with the RCT‐PPMM is that it does not directly correspond to parameterizing selection as dependent on the treatment effect itself, that is, on $Y^{1}-Y^{0}$. Selection dependent on the treatment effect is captured through having selection depend on one or both of the potential outcomes (but not explicitly their difference). An alternative but related approach would be to apply the PPMM directly to the difference $Y^{1}-Y^{0}$, thus directly modeling selection dependent on the treatment effect with a single ϕ parameter. The challenge with this approach, however, is that the two potential outcomes are never jointly observed, and thus the correlation between the potential outcomes is not identifiable. One potential alternative would be to include this correlation as a sensitivity parameter, along with a single ϕ parameter capturing how selection depends on the treatment effect. This approach is an avenue for future work.

At present, the RCT‐PPMM approach relies on an assumption of bivariate normality for the underlying PPMM. This limits the utility of the estimators proposed in this paper for outcome variables that are not continuous and/or are not approximately normal in distribution. Future extensions of this approach could leverage prior work [47] to develop adjusted estimators of treatment effects on proportions, and further extend this work to consider ordinal, multinomial, and count outcomes.

## Conflicts of Interest

The authors declare no conflicts of interest.

## Supporting information


**Data S1:** Supporting Information.

## Data Availability

The R code to perform the simulations is available at https://github.com/randridge/PPMA. The data from the yoga trial is not publicly available due to privacy restrictions.
