# Supplementary material for: A Sensitivity Analysis Framework Using the Proxy Pattern–Mixture Model for Generalization of Experimental Results
Source: Stat Med. 2025 Nov 7;44(25-27):e70313. doi: 10.1002/sim.70313 (PMC12593313; doi:10.1002/sim.70313)
Supplement: Supplementary file 1 — Data S1: Supporting Information. [file SIM-44-0-s001.pdf]

Supplemental Material for:

A Sensitivity Analysis Framework Using the Proxy Pattern-Mixture Model
for Generalization of Experimental Results

Pages 2-5: Supplemental Figures S1-S5

Pages 7-8: Supplemental Tables

Pages 9-12: Bias functions of Dahabreh et al. (2023) under the RCT-PPMM

Figure S1: Empirical mean maximum likelihood estimates (MLEs) of the treatment effect under the RCT-PPMM when there is a weak proxy ($\sigma^2 = 13$). Columns correspond to different selection (SEL) mechanisms and rows correspond to different effect modification (EM) scenarios, where selection/effect modification always depends on an unobserved effect modifier U and may also depend on observed covariates Z and/or W. True treatment effect is shown with a black vertical line, trial estimate and 95% confidence interval (constructed using the average standard error across replicates) is shown with a red triangle, and RCT-PPMM intervals based on the MLE point estimates are shown in green ($\phi_1 = \{0, 0.5, 1\}, \phi_0 = 0$) and blue ($\phi_1 = 0, \phi_0 = \{0, 0.5, 1\}$).

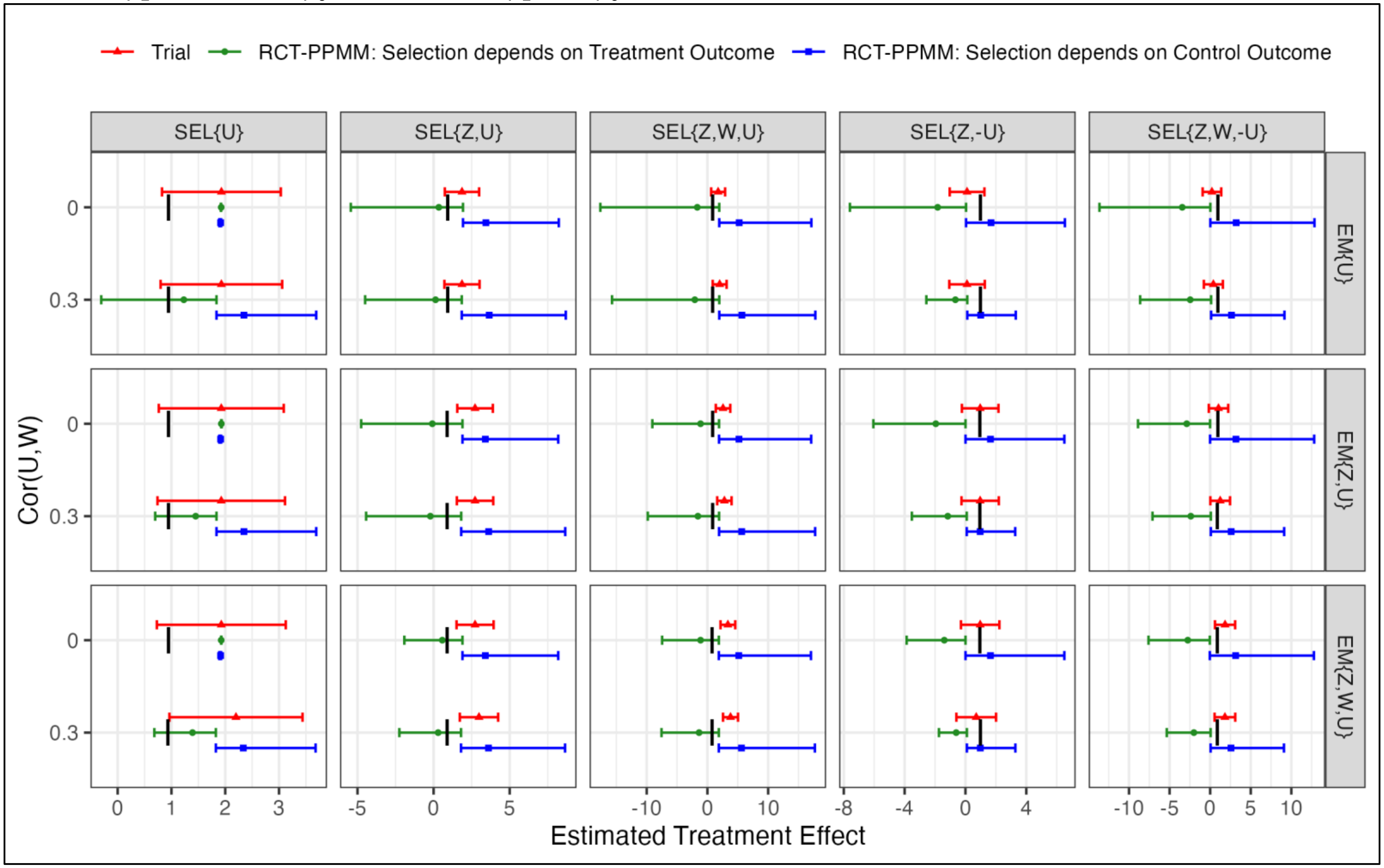

Figure S2: Empirical mean maximum likelihood estimates (MLEs) of the treatment effect under the RCT-PPMM when there is a strong proxy ($\sigma^2 = 1$). Columns correspond to different selection (SEL) mechanisms and rows correspond to different effect modification (EM) scenarios, where selection/effect modification always depends on an unobserved effect modifier U and may also depend on observed covariates Z and/or W. True treatment effect is shown with a black vertical line, trial estimate and 95% confidence interval (constructed using the average standard error across replicates) is shown with a red triangle, and RCT-PPMM intervals based on the MLE point estimates are shown in green ($\phi_1 = \{0, 0.5, 1\}, \phi_0 = 0$) and blue ($\phi_1 = 0, \phi_0 = \{0, 0.5, 1\}$).

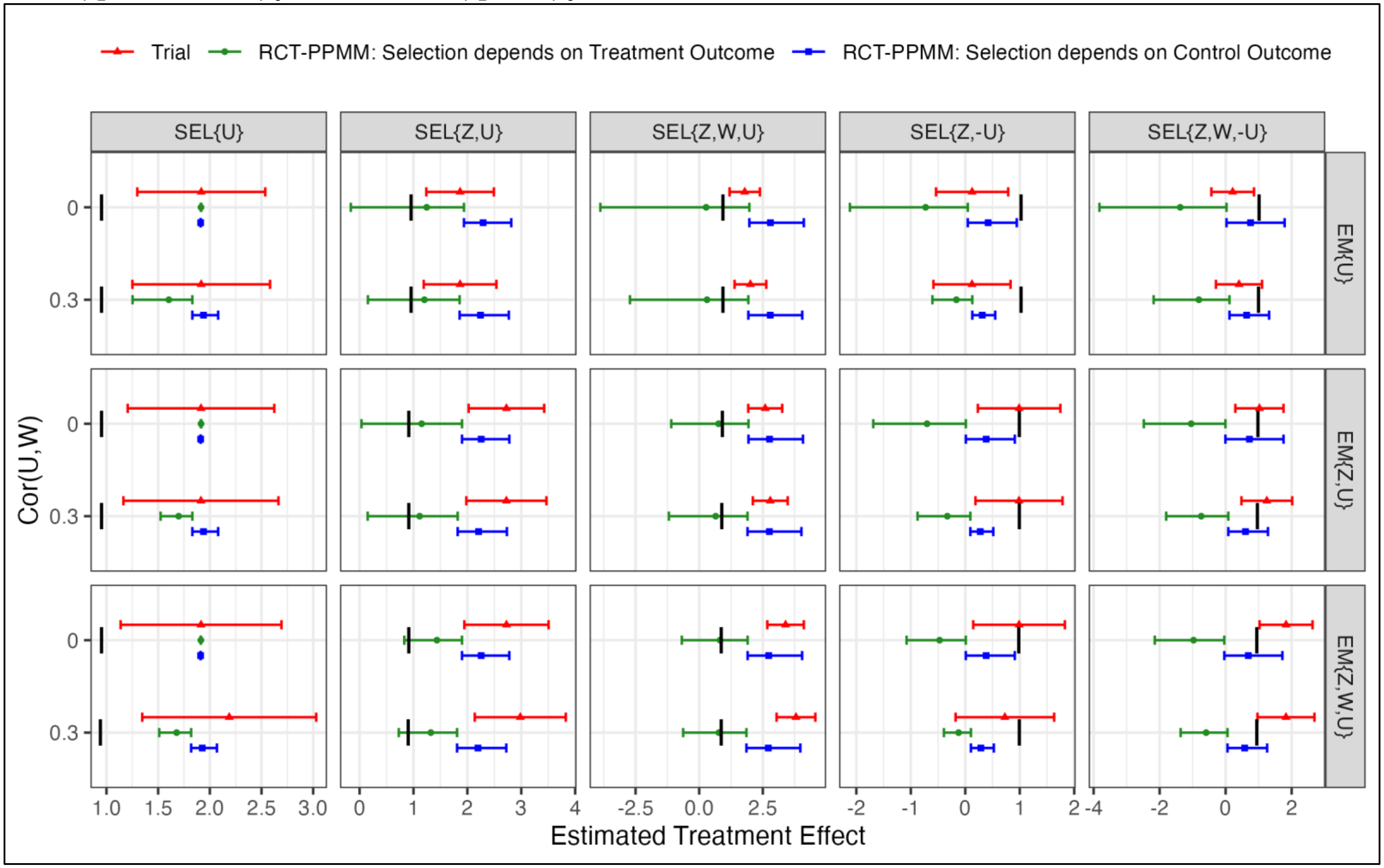

Figure S3: Maximum likelihood estimates (MLEs) under the RCT-PPMM for the fatigue outcome (MFSI-SF). Panels A and B show the estimated treatment effect (A) and group means (B) when selection depends on the control outcome $\{\phi_1 = 0, \phi_0 = \{0,0.1,0.2,\ldots,0.9,1\}\}$; Panels C and D show the estimated treatment effect (C) and group means (D) when selection depends on the treatment outcome $\{\phi_1 = \{0,0.1,0.2,\ldots,0.9,1\}, \phi_0 = 0\}$. Error bars show 95% confidence intervals with variance estimation by M-estimation.

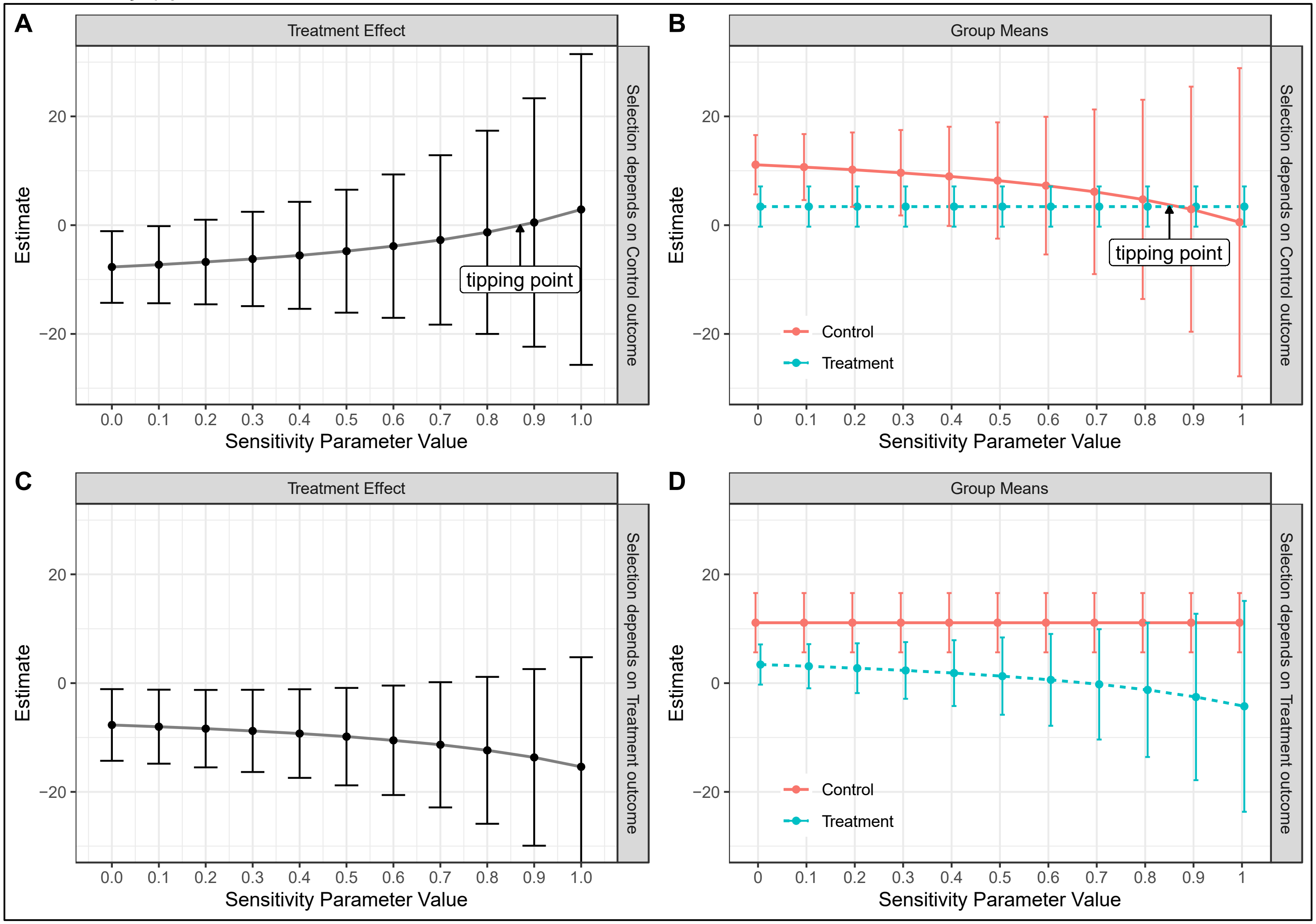

Figure S4: Maximum likelihood estimates (MLEs) under the RCT-PPMM for the vitality outcome (SF-36). Panels A and B show the estimated treatment effect (A) and group means (B) when selection depends on the control outcome $\{\phi_1 = 0, \phi_0 = \{0,0.1,0.2,\ldots,0.9,1\}\}$; Panels C and D show the estimated treatment effect (C) and group means (D) when selection depends on the treatment outcome $\{\phi_1 = \{0,0.1,0.2,\ldots,0.9,1\}, \phi_0 = 0\}$. Error bars show 95% confidence intervals with variance estimation by M-estimation.

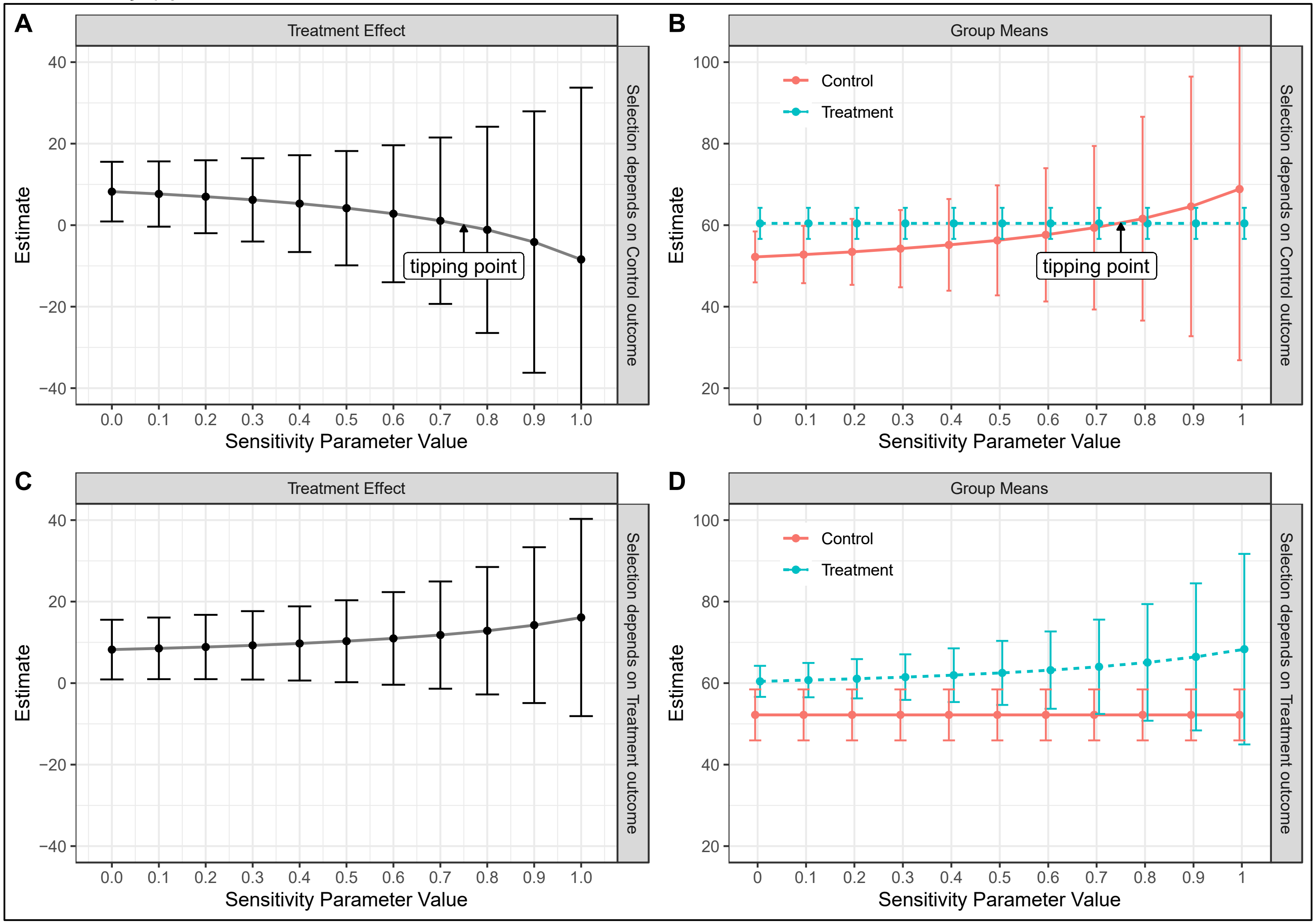

Figure S5: Bayesian estimates under the RCT-PPMM for the fatigue (MFSI-SF) and vitality (SF-36) outcomes when selection depends equally on the treatment and control outcomes ($\phi_1 = \phi_0$). Panels A and B show the estimated treatment effect (A) and group means (B) for fatigue; Panels C and D show the estimated treatment effect (C) and group means (D) for Vitality. Results are based on 10,000 posterior draws and shaded areas show 95% credible intervals.

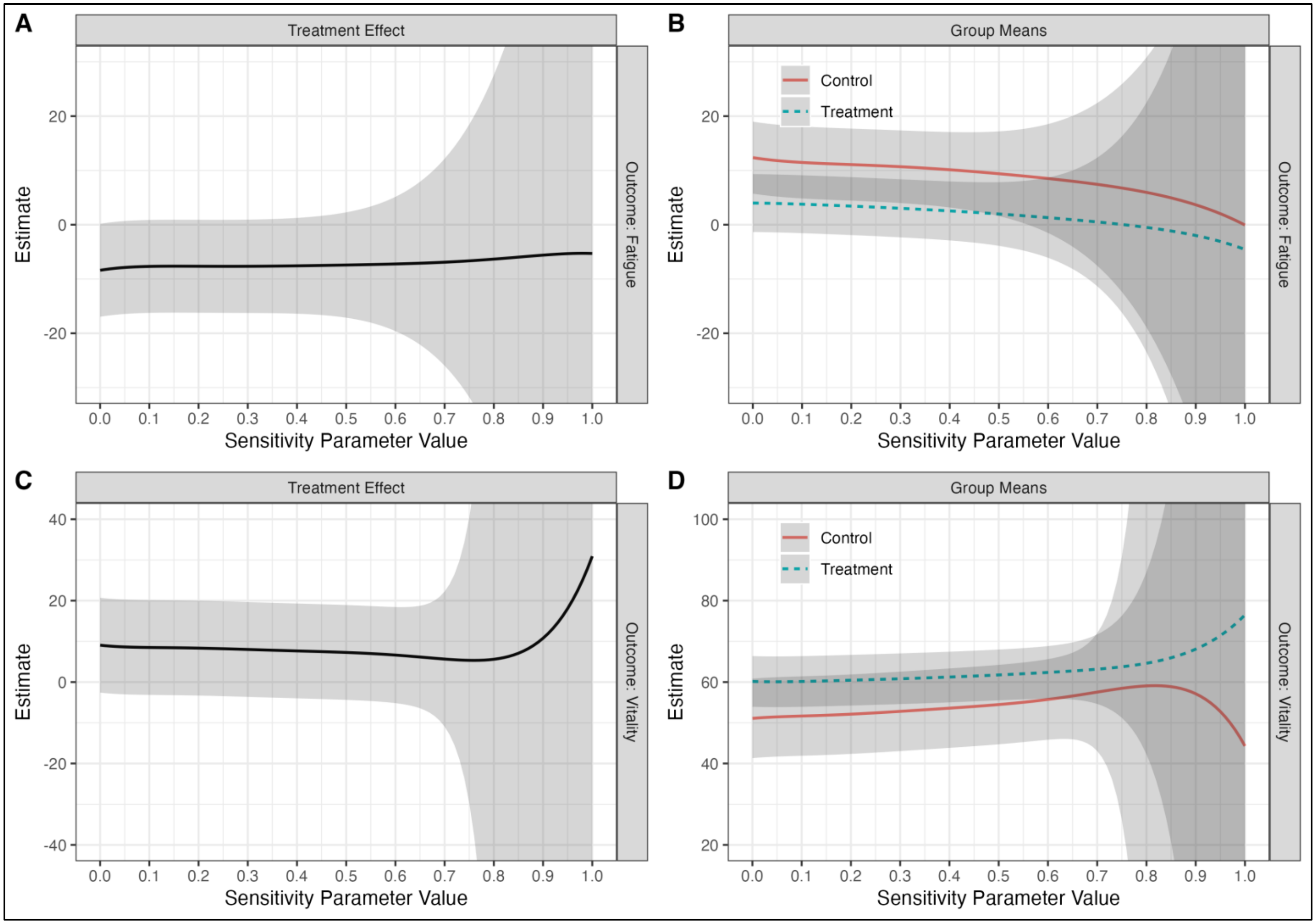

Table S1: Empirical coverage of intervals based on the RCT-PPMM for the simulation study when there is a strong proxy ($\sigma^2 = 1$).

			RCT-PPMM: Assume Selection Depends on Treatment Outcome			RCT-PPMM: Assume Selection Depends on Control Outcome		
**Selection depends on:**	**Effect modification by:**	**Corr(U, W)**	**MLE**	**Bayes / Uniform(0,1)**	**Bayes / Uniform(0,0.5)**	**MLE**	**Bayes / Uniform(0,1)**	**Bayes / Uniform(0,0.5)**
U	U	0	1.2	19.4	5.2	0.1	7.6	5.2
		0.3	21.4	65.0	22.8	0.0	4.8	6.6
	Z, U	0	0.1	9.7	4.7	0.1	7.3	5.2
		0.3	4.2	37.3	16.2	0.0	5.3	7.0
	Z, W, U	0	0.1	6.8	5.0	0.1	7.7	5.4
		0.3	2.9	34.9	16.2	0.0	5.2	7.1
Z, U	U	0	89.1	**98.4**	74.3	0.5	9.2	13.2
		0.3	89.7	**99.0**	75.3	0.7	10.9	17.4
	Z, U	0	**94.3**	**99.6**	73.9	0.5	9.8	13.3
		0.3	**94.6**	**99.6**	76.8	0.7	11.0	17.4
	Z, W, U	0	54.9	89.5	55.2	0.5	10.0	13.7
		0.3	63.3	**94.5**	63.8	0.7	12.1	18.3
Z, W, U	U	0	**98.4**	**99.9**	**99.6**	1.6	8.0	15.5
		0.3	**98.1**	**99.9**	**99.2**	1.9	13.0	21.5
	Z, U	0	**98.3**	**100.0**	93.3	1.6	9.3	15.7
		0.3	**98.0**	**99.8**	**95.6**	1.9	13.3	21.4
	Z, W, U	0	**98.3**	**100.0**	91.6	1.6	9.6	16.4
		0.3	**97.8**	**99.9**	92.0	1.9	13.5	22.3
Z, -U	U	0	0.1	3.6	8.9	40.8	83.2	44.9
		0.3	0.2	22.9	25.3	10.7	57.9	39.6
	Z, U	0	0.1	2.6	6.0	40.8	83.7	45.3
		0.3	0.2	11.4	17.5	10.7	59.5	40.3
	Z, W, U	0	0.1	4.5	9.2	40.8	83.7	44.8
		0.3	0.2	18.3	23.7	10.7	59.0	39.9
Z, W, -U	U	0	1.2	6.9	13.4	89.1	**99.0**	75.9
		0.3	0.9	7.6	14.2	72.7	**95.0**	68.0
	Z, U	0	1.2	7.8	15.4	89.1	**98.9**	74.4
		0.3	0.9	7.7	15.3	72.7	**94.8**	67.4
	Z, W, U	0	1.2	8.5	15.1	89.1	**98.8**	74.4
		0.3	0.9	9.4	17.3	72.7	**94.7**	67.9

Bold denotes empirical coverage at or above 95% (accounting for Monte Carlo simulation error)

Table S2: Empirical coverage of intervals based on the RCT-PPMM for the simulation study when there is a weak proxy ($\sigma^2 = 13$).

			RCT-PPMM: Assume Selection Depends on Treatment Outcome			RCT-PPMM: Assume Selection Depends on Control Outcome		
**Selection depends on:**	**Effect modification by:**	**Corr(U, W)**	**MLE**	**Bayes / Uniform(0,1)**	**Bayes / Uniform(0,0.5)**	**MLE**	**Bayes / Uniform(0,1)**	**Bayes / Uniform(0,0.5)**
U	U	0	21.7	82.1	64.0	19.0	78.4	63.6
		0.3	79.5	**99.2**	86.9	6.1	54.5	58.3
	Z, U	0	12.2	73.7	63.9	19.0	78.3	63.4
		0.3	53.1	**95.5**	82.1	6.1	55.1	58.1
	Z, W, U	0	9.6	72.1	61.9	19.0	78.9	64.0
		0.3	54.9	**95.0**	82.5	6.1	55.0	58.2
Z, U	U	0	84.2	**99.0**	**96.8**	9.6	57.4	61.8
		0.3	86.0	**98.5**	**98.4**	12.1	50.5	60.9
	Z, U	0	89.8	**99.0**	**99.2**	9.6	56.7	62.8
		0.3	87.7	**98.2**	**98.9**	12.1	51.0	61.8
	Z, W, U	0	87.6	**99.4**	**95.8**	9.6	58.3	63.5
		0.3	87.5	**98.6**	**98.3**	12.1	52.1	62.7
Z, W, U	U	0	84.2	**96.5**	**97.6**	14.7	47.1	55.9
		0.3	83.2	**94.6**	**98.1**	16.8	45.6	59.0
	Z, U	0	85.1	**96.6**	**97.5**	14.7	47.5	58.4
		0.3	83.2	**94.4**	**98.1**	16.8	46.5	59.9
	Z, W, U	0	85.1	**96.2**	**97.4**	14.7	49.0	57.7
		0.3	83.2	**94.4**	**98.0**	16.8	47.4	61.7
Z, -U	U	0	9.3	50.7	56.6	88.7	**98.5**	**97.7**
		0.3	10.5	74.1	73.9	70.6	**98.5**	**94.0**
	Z, U	0	9.6	36.2	48.7	88.7	**98.5**	**97.9**
		0.3	11.1	53.3	62.6	70.6	**98.4**	**94.6**
	Z, W, U	0	9.6	42.0	53.5	88.7	**98.9**	**97.4**
		0.3	8.9	62.9	68.8	70.6	**98.7**	**94.2**
Z, W, -U	U	0	14.6	40.3	53.7	85.4	**96.8**	**98.3**
		0.3	14.3	43.8	56.1	85.7	**96.7**	**98.0**
	Z, U	0	14.6	39.3	52.5	85.4	**97.0**	**98.7**
		0.3	14.3	40.7	53.8	85.7	**97.1**	**97.8**
	Z, W, U	0	14.6	37.6	51.4	85.4	**97.5**	**98.3**
		0.3	14.3	42.1	54.6	85.7	**97.3**	**97.9**

Bold denotes empirical coverage at or above 95% (accounting for Monte Carlo simulation error)

Reformulating the RCT-PPMM as the "bias functions" in Dahabreh et al. (2023)

We can reformulate the bias functions for sensitivity analysis proposed by Dahabreh et al. (2023) within our RCT-PPMM framework to investigate how our parameters (ϕ_1, ϕ_0) act within their proposed functions.

The bias function under the RCT-PPMM for each treatment arm $a \in \{0,1\}$ can be expressed as a function of (ϕ_1, ϕ_0), using equation (3) in the main paper:

$$u(a, \phi_a) = E[Y^a|S=1] - E[Y^a|S=0]$$

$$= \mu_{y(a)}^{(1)} - \mu_{y(a)}^{(0)} = \frac{\phi_a + (1-\phi_a)\rho_a^{(1)}}{\phi_a\rho_a^{(1)} + (1-\phi_a)}\sqrt{\frac{\sigma_{yy(a)}^{(1)}}{\sigma_{xx(a)}^{(1)}}}\left(\mu_{x(a)}^{(1)} - \mu_{x(a)}^{(0)}\right), \tag{1}$$

with simplified forms at $\phi_a = 0$ and $\phi_a = 1$:

$$u(a, \phi_a = 0) = \rho_a^{(1)}\sqrt{\frac{\sigma_{yy(a)}^{(1)}}{\sigma_{xx(a)}^{(1)}}}\left(\mu_{x(a)}^{(1)} - \mu_{x(a)}^{(0)}\right) \tag{2}$$

$$u(a, \phi_a = 1) = \frac{1}{\rho_a^{(1)}}\sqrt{\frac{\sigma_{yy(a)}^{(1)}}{\sigma_{xx(a)}^{(1)}}}\left(\mu_{x(a)}^{(1)} - \mu_{x(a)}^{(0)}\right). \tag{3}$$

The difference in the bias functions for the treatment and control arms, δ, can thus be expressed as a function of (ϕ_1, ϕ_0) as well:

$$\delta(\phi_1, \phi_0) = u(1, \phi_1) - u(0, \phi_0)$$

$$= \frac{\phi_1 + (1-\phi_1)\rho_1^{(1)}}{\phi_1\rho_1^{(1)} + (1-\phi_1)}\sqrt{\frac{\sigma_{yy(1)}^{(1)}}{\sigma_{xx(1)}^{(1)}}}\left(\mu_{x(1)}^{(1)} - \mu_{x(1)}^{(0)}\right) - \frac{\phi_0 + (1-\phi_0)\rho_0^{(1)}}{\phi_0\rho_0^{(1)} + (1-\phi_0)}\sqrt{\frac{\sigma_{yy(0)}^{(1)}}{\sigma_{xx(0)}^{(1)}}}(\mu_{x(0)}^{(1)} - \mu_{x(0)}^{(0)}) \tag{4}$$

The MLEs of the two bias functions used for the Dahabreh sensitivity analysis, $u(0)$ and δ, are then given by:

$$\hat{u}(0, \phi_0) = \frac{\phi_0 + (1-\phi_0)\hat{\rho}_0^{(1)}}{\phi_0\hat{\rho}_0^{(1)} + (1-\phi_0)}\sqrt{\frac{\hat{\sigma}_{yy(0)}^{(1)}}{\hat{\sigma}_{xx(0)}^{(1)}}}(\bar{x}_0^{(1)} - \bar{x}_0^{(0)}) \tag{5}$$

$$\hat{\delta}(\phi_1, \phi_0) = \frac{\phi_1 + (1-\phi_1)\hat{\rho}_1^{(1)}}{\phi_1\hat{\rho}_1^{(1)} + (1-\phi_1)}\sqrt{\frac{\hat{\sigma}_{yy(1)}^{(1)}}{\hat{\sigma}_{xx(1)}^{(1)}}}(\bar{x}_1^{(1)} - \bar{x}_1^{(0)}) - \frac{\phi_0 + (1-\phi_0)\hat{\rho}_0^{(1)}}{\phi_0\hat{\rho}_0^{(1)} + (1-\phi_0)}\sqrt{\frac{\hat{\sigma}_{yy(0)}^{(1)}}{\hat{\sigma}_{xx(0)}^{(1)}}}(\bar{x}_0^{(1)} - \bar{x}_0^{(0)}) \tag{6}$$

We consider two combinations of the sensitivity parameters: selection dependent on the potential outcome under treatment $\{\phi_1$ varies, ϕ_0 fixed to zero$\}$, and selection dependent on the potential outcome under control $\{\phi_1$ fixed to zero, ϕ_0 varies$\}$. For each scenario, we show the pair of bias functions used in the Dahabreh approach $\{u(0), \delta\}$ under the RCT-PPMM.

Selection dependent on the outcome under treatment

When selection is dependent on the outcome under treatment, we vary ϕ_1 and fix ϕ_0 to zero. Thus, based on equation (5), $\hat{u}(0, \phi_0 = 0)$ is constant with respect to ϕ_1:

$$u(0, \phi_0 = 0) = \hat{\rho}_0^{(1)} \sqrt{\frac{\hat{\sigma}_{yy(0)}^{(1)}}{\hat{\sigma}_{xx(0)}^{(1)}}} \left(\bar{x}_0^{(1)} - \bar{x}_0^{(0)}\right)$$

and based on equation (6), $\hat{\delta}\left(\phi_1, \phi_0 = 0\right)$ is a function of ϕ_1. Thus, the RCT-PPMM analysis assuming selection depends on treatment corresponds to setting the bias function for the control group, $u(0)$, to a data-driven fixed estimate, and varying δ.

Using data from the yoga intervention RCT we can plot the estimated treatment effect and group means as a function of the δ, noticing that δ is bounded by the bounding of $\phi_1 \in [0,1]$:

Figure 1 Sensitivity analysis results for Fatigue when selection depends on the treatment outcome.

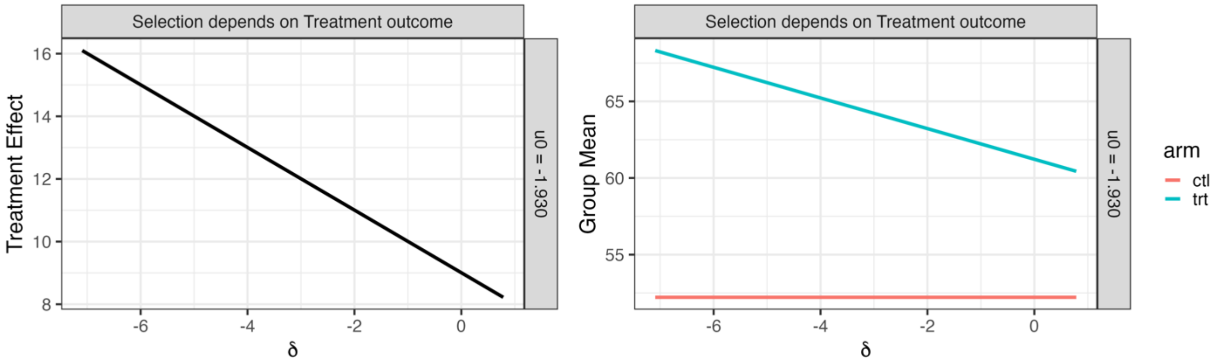

Figure 2 Sensitivity analysis results for Vitality when selection depends on the treatment outcome.

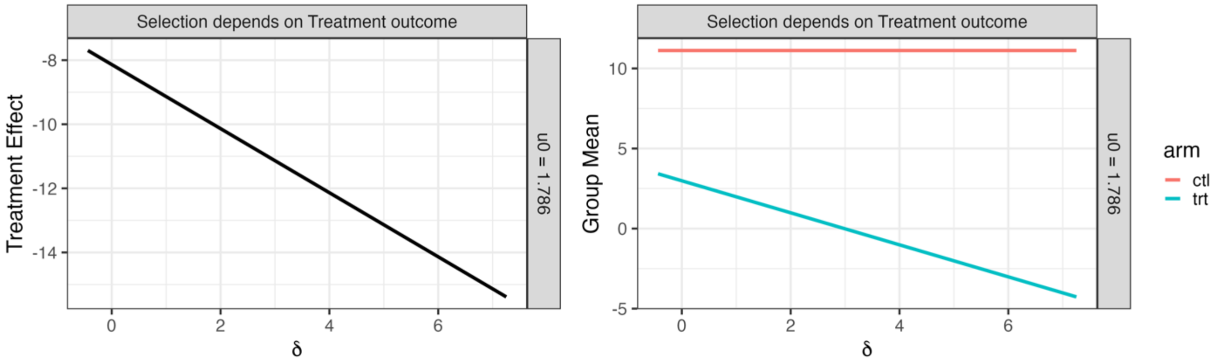

Selection dependent on the outcome under control

When selection is dependent on the outcome under control, we fix ϕ_1 to zero and vary ϕ_0. As a result, $u(0)$ is not a fixed quantity but rather depends on ϕ_0 (as in equation (5)), and δ also is a function of ϕ_0. Thus, for the sensitivity analysis we can either vary $u(0)$ or δ but not both, as they are both fixed for a given ϕ_0.

Using data from the yoga intervention RCT, we can plot the estimated treatment effect and group means as a function of the δ or as a function of $u(0)$, with both quantities bounded by the bounding of $\phi_0 \in [0,1]$:

Figure 3 Sensitivity analysis results for Fatigue when selection depends on the control outcome.

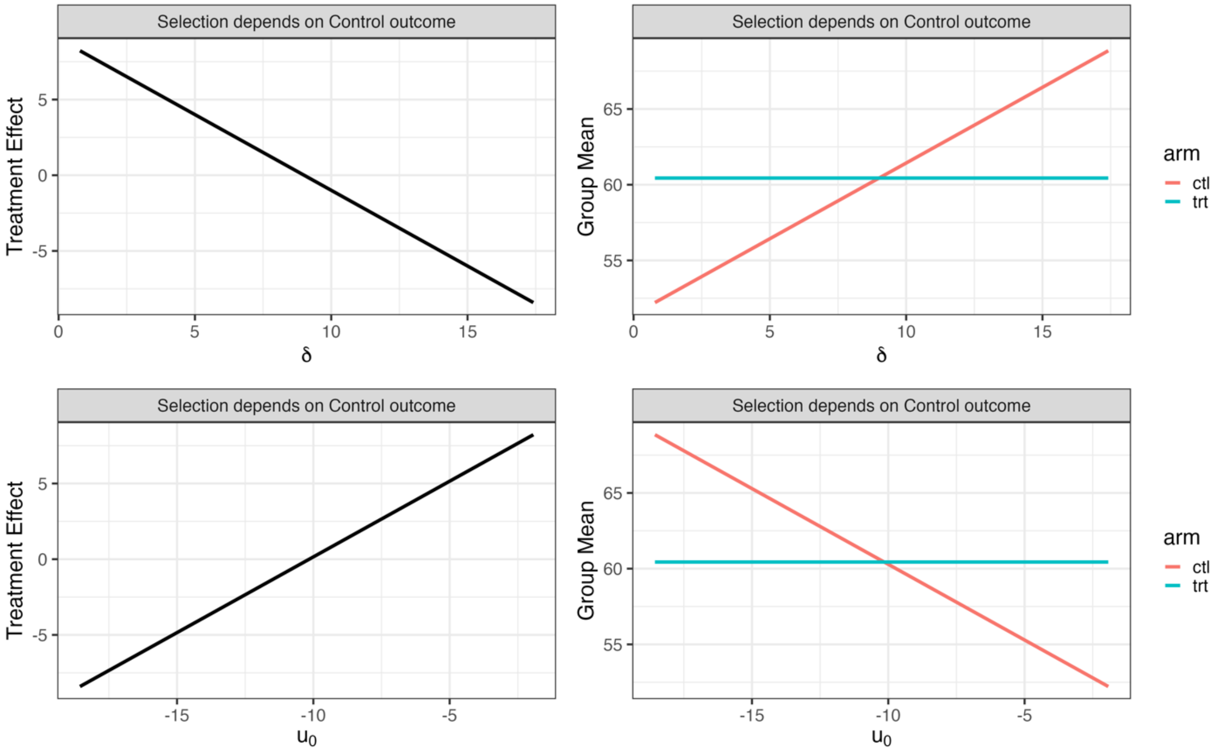

Figure 4 Sensitivity analysis results for Vitality when selection depends on the control outcome.

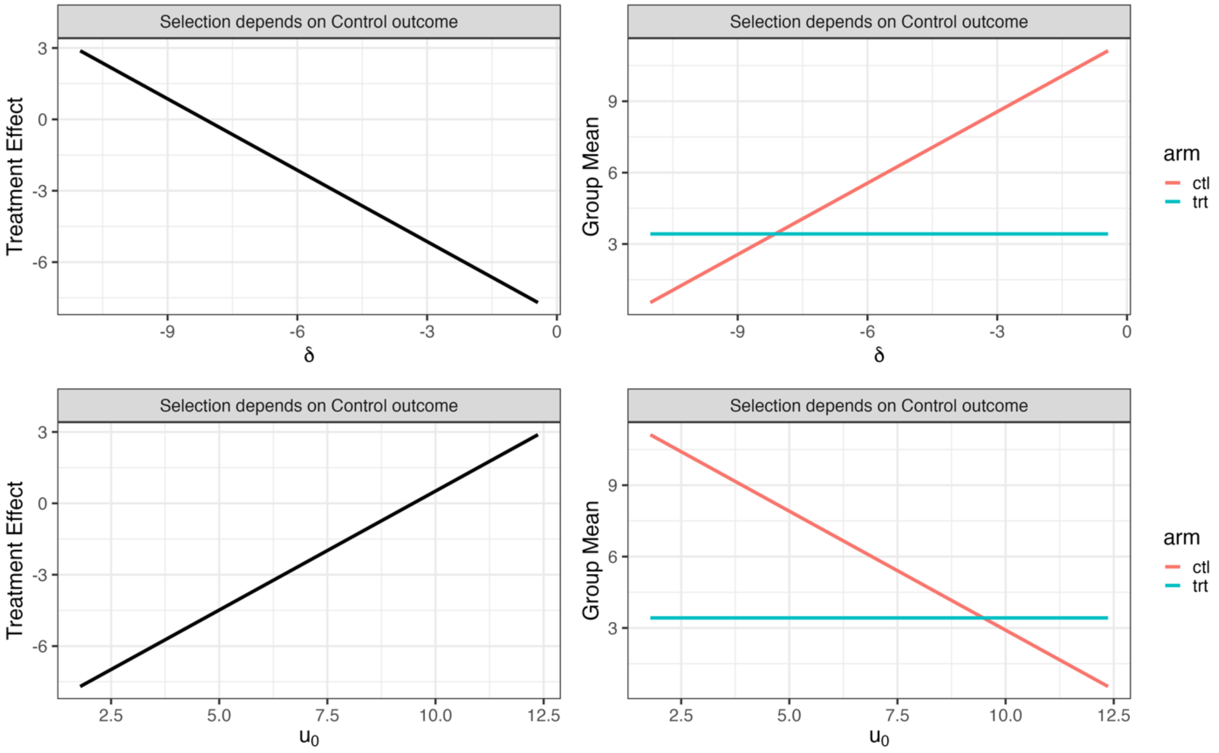

Reference

Dahabreh IJ, Robins JM, Haneuse S, et al. Sensitivity analysis using bias functions for studies extending inferences from a randomized trial to a target population. *Statistics in Medicine*. 2023;42(13):2029-2043. doi:10.1002/sim.9550
